# Towards affordable CRISPR genomic therapies: a task force convened by the Innovative Genomics Institute

**DOI:** 10.1038/s41434-023-00392-3

**Published:** 2023-11-08

**Authors:** Lea Witkowsky, Matthew Norstad, Audrey R. Glynn, Melinda Kliegman

**Affiliations:** 1 University of California, Berkeley, Kavli Center for Ethics, Science, and the Public, 621 Sutardja Dai Hall, Berkeley, CA 94720 USA; 2 University of California, San Francisco, Bioethics Program, 490 Illinois Street, 12th Floor, San Francisco, CA 94143 USA; 3 University of California, Berkeley, Innovative Genomics Institute, 2151 Berkeley Way, Berkeley, CA 94720 USA

**Keywords:** Targeted gene repair, Targeted gene repair, Genetic engineering, Drug delivery

## Introduction

For individuals living with debilitating hereditary diseases, therapies that can modify a person’s genome (genomic therapies) hold the promise to shift treatment outlooks from a lifetime of chronic disease management to a cure [1]. We now have groundbreaking therapies for multiple disorders, including genetic eye diseases, spinal muscular atrophy, and beta-thalassemia (Table 1). Additional genomic therapies targeting other hemoglobinopathies and immune deficiencies are currently under evaluation in clinical trials [2].

**Table 1:** These 12 genomic therapies were selected from the FDA’s list of 27 approved cellular and gene therapy (CGT) products, as of December, 16, 2022.

Therapy	Date Approved	Drug Description	Disease	Price
**KYMRIAH** - Novartis Pharmaceuticals Corporation	8/30/2017	CD19-directed autologous CAR T-cells (ex vivo)	follicular lymphoma	$475,000 [28]
**YESCARTA** - Kite Pharma, Inc. (Gilead)	10/18/2017	CD19-directed autologous CAR T-cells (ex vivo)	large B-cell lymphoma, a type of non-Hodgkin lymphoma	$373,000 [29]
**LUXTURNA** - Spark Therapeutics, Inc.	12/18/2017	AAV carrying a functional RPE65 gene (in vivo)	biallelic RPE65 mutation-associated retinal dystrophy.	$425,000 [30]
**ZOLGENSMA** - Novartis Gene Therapies, Inc.	5/24/2019	AAV carrying a functional SMN1 gene (in vivo)	spinal muscular atrophy	$2.125 M [31]
**TECARTUS** - Kite Pharma, Inc. (Gilead)	7/24/2020	CD19-directed autologous CAR T-cells (ex vivo)	mantle cell lymphoma or acute lymphoblastic leukemia	$373,000 [32]
**BREYANZI** - Juno Therapeutics, Inc., a Bristol-Myers Squibb Company	2/5/2021	CD19-directed autologous CAR T-cells (ex vivo)	large B-cell lymphoma	$410,300 [33]
**ABECMA** - Celgene Corporation, a Bristol-Myers Squibb Company	3/26/2021	BCMA-directed autologous CAR T-cells (ex vivo)	multiple myeloma	$419,500 [34]
**CARVYKTI** - Janssen Biotech, Inc.	2/28/2022	BCMA-directed autologous CAR T-cells (ex vivo)	multiple myeloma	$465,000 [35]
**ZYNTEGLO** - bluebird bio, Inc.	8/17/2022	Autologous HSP cells modified with a LVV carrying a form of the ß-globin gene (ex vivo)	ß-thalassemia	$2.8 M (USA) [36]$1.8 M* (EU)
**SKYSONA** - bluebird bio, Inc.	9/16/2022	Autologous HSP cells modified with a LVV carrying a functional ABCD1 gene (ex vivo)	cerebral adrenoleukodystrophy	$3.0 M [37]
**HEMGENIX** - CSL Behring LLC	11/22/2022	AAV carrying a form of the factor IX gene (in vivo)	Hemophilia B	$3.5 M [38]
**ADSTILADRIN** - Ferring Pharmaceuticals A/S	12/16/2022	AAV carrying a functional interferon alfa-2b gene (in vivo)	Non-Muscle Invasive Bladder Cancer	not available

Only those FDA-approved therapies that derive their therapeutic effect as a result of modification to the human genome, including addition of a transgene, are included in this list. Imlygic (talimogene laherparepvec) from BioVex, Inc. was excluded as it did not squarely fit the criteria. Date approved corresponds to the date of the FDA’s BLA approval letter. The drug description includes whether the genomic modification is executed in vivo or ex vivo.

*CAR* chimeric antigen receptors, *AAV* adeno-associated virus, *HSP* hematopoietic stem and progenitor, *LVV* lentiviral vector.

*After EMA approval and price setting, Bluebird Bio pulled out of the EU.

Unfortunately, the first generation of genomic therapies have entered the market with price tags up to $3.5 million per treatment (Table 1), with reports of insurance companies denying or delaying coverage to qualified individuals [3]. These price tags threaten the financial stability of insurance markets—particularly public markets, such as Medicaid and government funded healthcare systems in Europe - as well as the ability of patients to access the therapies. By one estimate, if a sickle cell disease (SCD) genomic therapy came to market at $1 million per patient, it would cost Medicaid $55 billion or roughly 85% of Medicaid’s total spending on outpatient drugs in 2017 [4]. These prices are often justified on the basis that genomic therapies are one-time, potentially curative treatments that offer high value to patients and health systems by negating future costs related to treating the disease (Box 1).

For diseases disproportionately affecting marginalized communities (e.g., SCD primarily affects individuals of African ancestry and Artemis Severe Combined Immunodeficiency (SCID) is highly prevalent in Navajo and Apache communities [5]), affordability and accessibility are matters of health equity.

Even more troubling are signs that traditional for-profit pharmaceutical companies may be unsuitable for delivering genomic therapies for rare diseases. Orchard Therapeutics secured a license to commercialize a gene therapy for SCID but returned the license to the University of California due to manufacturing issues [6]. Bluebird bio developed SCD and beta thalassemia therapies with positive regulatory determinations, but shut down operations in Europe citing “challenges of achieving appropriate value recognition and market access” [7].

In a for-profit context, deprioritization of genomic therapies for rare diseases is logical because commercialization is only feasible if the economics are positive. With small patient populations, the revenue expected from rare genomic therapies, even when highly priced, may be insufficient for commercial entities to justify the considerable upfront investment [8]. Given the difficulties pharmaceutical companies face to advance clinically successful genomic therapies for rare diseases, viable alternative models of production are needed to meet the promise of this technology.

To investigate the underlying drivers of high prices and explore alternative pathways to development, the Innovative Genomics Institute (IGI) launched an expert Affordability Task Force in January 2022. The 35-member task force is evaluating business models and funding, manufacturing challenges, intellectual property, health insurance and health policy. While the task force’s work will not be completed until early 2023, here we present its early findings.

Box 1 Value based pricing and payment models Value-based pricing attempts to reconcile prices with the value provided to patients and health systems, and thus creates an added incentive to produce highly effective drugs. A push for value-based pricing and payment models has been observed in recent years as overpriced, under-performing drugs, like Aduhelm for Alzheimer’s, draw public scrutiny [39]. However, when high prices, set by private entities, capture the entirety of the estimated social value of a product, little remains to be shared with the health system or patients. More importantly, high prices do not guarantee equitable distribution.

In a purely value-based pricing system, reduced drug production costs are unlikely to significantly reduce prices since the value of a drug is wholly separate from input costs. Technological improvements that lower development costs, may increase profits for companies while not improving affordability for patients. Thus, value-based payment models [40] do not sufficiently resolve access and affordability.

### The current system

**Fig. 1:**
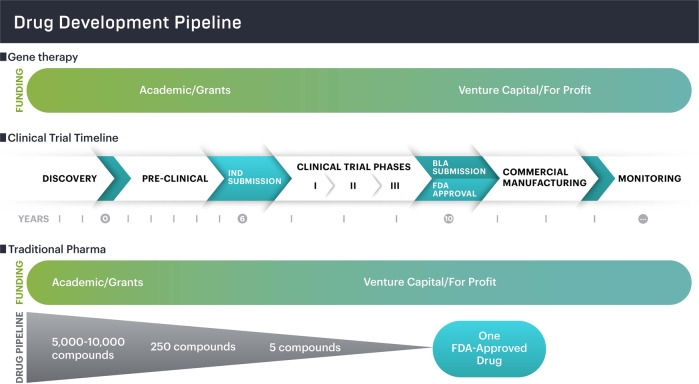
Pipeline for genome therapies and traditional pharmaceuticals. In the development of traditional pharmaceutical drugs, many compounds are eliminated from the development pipeline as companies move towards clinical trials and a commercializable product. The high failure rate of drugs in pre-clinical and early phase trials often justifies high prices upon approval because companies must recoup costs for failed drugs. Given the increase in state and federal funding of genome therapies, drug development risk has shifted from private, for-profit companies to the public.

The path to market for new drugs has remained virtually unchanged for decades: governments and philanthropies fund early-stage research at academic institutions with the technology then licensed by for-profit companies who further develop, clinically evaluate, and bring drug candidates through regulatory approvals. These companies take on the risk of failure at the clinical and regulatory stages. (Fig. 1) [9].

Today, an increasing number of government and philanthropy programs are challenging this traditional pipeline by not only funding early-stage research, but also funding clinical trials and providing regulatory support to accelerate promising new drugs [10]. This model reduces drug development risks for companies developing genomic therapies. However, even when research, development, and clinical trials are de-risked at academic or nonprofit organizations, this greater public investment does not guarantee lower market prices after drug candidates are licensed to for-profit companies. Private companies have a fiduciary responsibility to maximize profits for shareholders, and recent analyses indicate that increased profits are not reinvested in research and development [11] but instead are distributed to shareholders via stock buybacks [12, 13].

## Approach

To address this challenge, the IGI has assembled an expert Affordability Task Force composed of interdisciplinary scholars and practitioners with insights into all aspects of drug development and healthcare economics. Working with the IGI team, the task force is generating a roadmap of concrete strategies to increase the likelihood that genomic therapies developed by the IGI and other academic institutions are affordable and accessible [14]. We aim to shift from conversation to action by convening the right experts and charging them with creating practical solutions.

### Affordability Task Force design

**Fig. 2:**
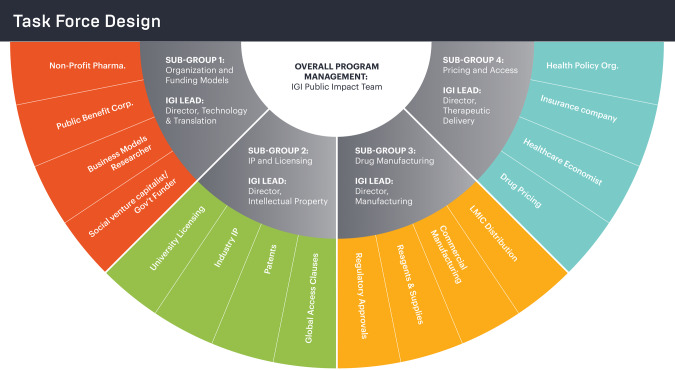
Affordability Task Force design. Experts across multiple disciplines and sectors (colored wheel slices) were divided into sub-groups (gray sections) focused on topical areas pertinent to envisioning new models to bring therapies to patients more affordably. Each sub-group was led by a member of IGI’s leadership and the project was managed by the IGI’s public impact team (white center).

The task force is divided into four sub-groups addressing specific aims critical to affordability and accessibility (Fig. 2). The first is evaluating alternative organizational models and how those organizations (including public benefit corporations, nonprofits, and government-run organizations) may operate sustainably. The second sub-group is focusing on the influence of patents, university licensing systems, and global access clauses. The third is focusing on manufacturing costs in alignment with regulatory requirements. The fourth sub-group is creating alternative pricing models, determining how such processes would operate in the broader healthcare system in terms of public and private insurance coverage [15], and elucidating the roles of hospitals, pharmacy benefit managers, and public policy in price determination. Facilitated and supported by IGI staff, sub-groups convene monthly to review literature, conduct interviews, and draft findings. This structure ensures experts can discuss complexities related to their specific areas of expertise; the entire task force also meets monthly to share findings and discuss cross-cutting issues.

## Early findings

To the best of our knowledge, this effort represents the most comprehensive attempt to understand and develop solutions to the affordability crisis facing new genomic therapies.

### Organization and funding models

The current drug development system is centered on commercialization tied to for-profit pharmaceuticals. However, real-world examples of alternative organizational models have emerged in recent years that exemplify different incentive structures. First passed into law in 2010 in Maryland, a Public Benefit Corporation is a type of for-profit corporate entity legally permitted to consider social goals rather than only shareholder value, and may be well-suited for drug development. United Therapeutics and Audacity Therapeutics are already using this model for drug development, relying on investors with a shared social vision, such as venture philanthropy.

Nonprofit drug organizations, like CivicaRx, have also emerged as potential models. In 2018, a group of health systems and philanthropies founded and financed CivicaRx to address generic drug pricing and shortage issues. Instead of separating investors and customers, CivicaRx is pioneering a Health Care Utility Model where the purchasers of the products fund the company [16]. This model eliminates middlemen, guarantees large volumes at a fixed price, secures hospital supply, and guarantees a steady income stream to the producer.

Organizational models focusing on the role of government and public funds may also be an effective approach. Frustrated by prices preventing access to necessary drugs, the state of California recently allocated $100 million to acquire and manufacture an insulin biosimilar with the goal of providing low-cost insulin to residents [17]. Separately, California voters twice passed propositions to fund the California Institute of Regenerative Medicine (CIRM) through bonds to finance the development of regenerative medicines such as genomic therapies. Since its creation in 2004, CIRM has funded over 80 clinical trials, created the Alpha Clinics Network for clinical trials, and led to over 50 startup companies with roots in CIRM-funded research projects [18]. This public funding investment has significantly stimulated and financially derisked the creation of these new therapies.

Although CIRM supports genomic therapies, it functions more as a funder than an organization that creates and distributes drugs. Most examples of alternative models for commercialization or distribution of drugs focus on generics or repurposed pharmaceuticals. Compared to these small molecule drugs, genomic therapies represent cutting edge technologies with valuable IP and a complex manufacturing process. Existing alternative models may need to be adapted to consider the unique properties of genomic therapies. A model that leverages the existing ecosystem of public dollars, grants, and philanthropy could make use of the unique expertise and manufacturing capacity of academic medical centers to create centers of excellence through strategic partnerships and operate under a nonprofit or public benefit designation.

### Intellectual property and licensing

To ensure affordability and access, universities, particularly those with a public mission, should consider integrating affordability and accessibility clauses into licensing agreements along with tracking and enforcement mechanisms. Resources such as the Master Alliance Provisions Guide (MAPGuide) [19] and Medicines Patent Pool (MPP) [20] were created to lower the barrier for developing agreements that uphold these values. The Global Healthcare Innovation Alliance Accelerator has combed through mountains of global drug and health agreements obtained through SEC filings and other submissions to identify examples of individual provisions that seek to increase global access, attenuate pricing, share benefits and more, and turned this analysis into an online toolkit in the MAPGuide.

While pricing and access are concerns for genomic therapies here in the US and in other high income countries, these concerns are compounded when considering whether and how these therapies will reach low and middle income countries. Medicines Patent Pool (MPP) was created to address this very type of challenge through non-exclusive voluntary licensing. MPP negotiates and manages licensing pools for IP, which makes achieving equitable access easier for patent holders who wish to reach a more global market but are not prepared to develop manufacturing and distribution infrastructure in developing countries on their own. The effectiveness of this approach was recently published, showing that upon inclusion in MPP, patents see “an immediate and large increase in licensing” and lead to an increase in product sales [21].

In addition to non-exclusive licensing, universities could include provisions in exclusive licenses that request or mandate companies to provide an agreed upon number of doses to low-income individuals, provide the product or sub-license the technology at reduced cost for those serving low and middle income countries, or require the creation of an accessibility and affordability plan with mechanisms for enforcement such as claw-back provisions.

### Manufacturing and regulations

Manufacturing costs and compliance with US regulations present a major bottleneck. The cost of building a dedicated current good manufacturing practice (cGMP) facility for a specific drug product is on the order of billions of dollars and any organization pursuing this approach needs significant upfront capital investment. Distributed manufacturing models may lower the cost of cell-based therapies [22] and have had some success in countries with single payer systems like Canada, where there are two major clinical trials using point-of-care manufacturing platforms for CAR-T cell products for infusion. The “Made-in-Canada CAR-T” platform cell manufacturing program uses a GMP-enabled manufacturing device to produce a cell therapy dose in 7–10 days, and it has already been used for 50 patients at less than 1/10 of the cost of a commercial product (e.g., $40,000 vs. $475,000 per dose of CD19 CAR-T for certain cancer patients) [23]. Of the total $15 M investment to create the Made-in-Alberta platform, the Alberta provincial government contributed $10 M and the Alberta Cancer Foundation contributed $5 M to initiate the clinical trial. The Government of Canada has also contributed significant funding to infrastructure and clinical trials support to promote cell and gene therapy manufacturing in Canada at point-of-care. It is anticipated that the $15 M investment will be recovered within 6 months of rollout (i.e., approved product rather than clinical trial) in Alberta.

Despite all its advantages, point-of-care manufacturing models are difficult to implement in the US given FDA requirements. Lowering the cost of CRISPR-based therapies may necessitate regulatory streamlining. One proposed path is to minimize requirements for cGMP grade reagents in investigational new drug (IND)-enabling and clinical studies. In the context of editing autologous cells, the Cas protein and guide RNA become diluted and degraded before the cell product is returned to the patient [24, 25]. These reagents could be manufactured at research grade while the final modified cells are held to cGMP standards. Alternatively, the FDA could regulate CRISPR-based therapies by approving broad platform technologies where possible instead of requiring a separate IND for every indication, and thereby leverage CRISPR modularity to accelerate approval and lower the cost of new therapies generated by the same therapeutic platform.

Advances in manufacturing, delivery, and regulatory frameworks will be necessary to lower the cost of goods for genomic therapies and increase potential for administration, but these advances are insufficient if they are not paired with mechanisms to lower prices and create new models of commercialization (see Box 1).

### Pricing and access

Conventional approaches to pricing—based on what the market can bear—have led to historically high prices. Most alternative methods identified by our Task Force aim to lower prices by tethering the final price of the product to the cost to develop and deliver the drug. Simple cost-plus models that calculate the cost of goods, labor, plus some predetermined profit margin are one approach that have been discussed in the literature [26]. The primary benefit of these models is transparency, and assurance that technological improvements will lower costs throughout the system. Such models have recently received popular press through the financier Mark Cuban who has launched his own Cost Plus Drug Company as a way to bring generic prescription drugs to market [27].

Despite their benefits, simplified cost-plus approaches have drawbacks that prevent organizations from easily adapting to market systems unique to innovative genomic therapies. Our task force members are developing a dynamic pricing model that includes the cost of drug development, number of patients requiring treatment, insurance coverage, market conditions, and the cost of capital. Under various modeling scenarios this dynamic cost-plus model yielded prices significantly less, in some cases up to 10x less than genomic therapies currently on the market, whilst still supporting a sustainable business.

Any new low-cost genomic therapy also needs to fit into the larger healthcare ecosystem. In some cases, misaligned incentives within the system may lead to higher prices for patients; for example, pharmacy benefit managers and hospitals are paid a percentage of the total cost of administering a drug, therefore drugs priced significantly lower than a competitor’s may be unattractive. Models such as CivicaRx may help address some of these issues through direct partnerships with health systems. Any entity seeking to establish a new/alternative structure will need to consider all the major healthcare system players (e.g., hospitals, insurance companies, and benefit managers) and ideally negotiate contracts prior to release to ensure a smooth transition and guarantee market share.

## Conclusion

Concerns over cost and distribution of benefits have been at the forefront of every debate since CRISPR genome editing was first developed, yet no substantive action has occurred. This project takes a first step toward developing a practical roadmap for academic institutions as the engines of genomic innovation.
